# Rescue of self-reactive B cells by provision of T cell help *in vivo*

**DOI:** 10.1002/(SICI)1521-4141(199808)28:08<2549::AID-IMMU2549>3.0.CO;2-O

**Published:** 1998-12-14

**Authors:** Matthew C Cook, Antony Basten, Barbara Fazekas de St. Groth

**Affiliations:** Centenary Institute of Cancer Medicine and Cell BiologyNewtown, Australia

**Keywords:** B cell, Tolerance, T cell help

## Abstract

We have previously demonstrated that antigen-specific T cell help can rescue mature Ig transgenic (Tg) hen egg lysozyme (HEL)-specific B cells from tolerance induction upon transfer into soluble HEL-expressing Tg hosts. Here we extend these findings by showing that T cell help could also rescue both immature and mature self-reactive B cells from rapid deletion in response to high-avidity membrane-bound HEL. Moreover, although short-lived anergic peripheral B cells that had matured in the presence of soluble self antigen could not be rescued by provision of T cell help, a proportion of immature anergic IgM^+^ IgD^−^ CD23^−^ B cells from the bone marrow of the same donors survived and proliferated when given help following transfer to a soluble or membrane HEL-expressing host. In other words, T cell help must be available relatively soon after the antigen signal to prevent induction of tolerance. Consistent with this interpretation, the stronger stimulus provided by membrane-bound antigen, which deletes immature B cells before they leave the bone marrow, did not afford an opportunity for T cell help to rescue tolerant immature bone marrow-derived B cells upon transfer *in vivo.* Nevertheless, these B cells were capable of responding to T cell help *in vitro,* which speaks against an immutable susceptibility of immature B cells to tolerance induction. Taken together, these data indicate that the strength of the antigen signal and availability of T cell help are the primary determinants of the fate of both immature and mature B cells, consistent with the model proposed by Bretscher and Cohn more than 25 years ago.

## 1 Introduction

Bretscher and Cohn were the first to propose that the response of an antigen-specific lymphocyte might be determined by an interaction with a second antigen-specific cell [1]. Their original model postulated that “antibody induction involves obligatory recognition of two determinants of an antigen by different antibody molecules”. The second antibody, termed “carrier antibody”, has now generally been replaced by T cell help in modern revisions of the theory. The Bretscher-Cohn model solved many of the difficulties of the previous models of Burnet and colleagues [2, 3] and Lederberg [4], which proposed a window of obligatory tolerance induction in the ontogeny of the animal [2, 3] or of each individual lymphocyte [4]. While the experimental evidence indicates that immunity can be induced early in ontogeny [5–9] and tolerance can be induced in the periphery of adult animals [10], thus formally refuting the original models, the idea that immature lymphocytes are predisposed to tolerance has remained a powerful concept in the field of self tolerance.

In the well-characterized hen egg lysozyme (HEL)-specific Ig transgenic (Tg) model [11], exposure of naive B cells to self antigen by adoptive transfer into a host expressing HEL as a neo-self antigen induces tolerance which is indistinguishable from that observed in mice co-expressing both HEL and the anti-HEL B cell receptor (BCR) (double Tg mice). Both immature (IgM^+^ IgD^−^ CD23^−^) bone marrow B cells [12] and mature (IgM^+^ IgD^+^) splenic B cells [13] are susceptible to tolerance induction and display indistinguishable sensitivity to the tolerogenic signals of different avidity provided by a range of concentrations of soluble HEL (sHEL) [13, 14]. We have recently demonstrated that provision of T cell help can rescue mature HEL-specific B cells from induction of tolerance to sHEL in this model [12, 15]. Thus, our experimental evidence is consistent with the Bretscher-Cohn hypothesis. On the other hand, attempts to rescue short-lived peripheral B cells which have matured in the presence of soluble self antigen have been unsuccessful in most cases [16, 17], although in two instances, the combination of primed or alloreactive T cells and a strong antigen stimulus appeared to permit rescue [18, 19]. These data raise the possibility that the requirements for rescue of B cells tolerized at an immature stage are more stringent, suggesting a developmental component in sensitivity to tolerance induction by self antigen.

To understand the factors responsible for regulating induction of self tolerance in B cells at different stages in their development, the responsiveness of immature and mature anti-HEL Tg B cells provided with antigen-specific T cell help was compared following recognition of self antigen *in vivo.* Immature (IgM^+^ IgD^−^ CD23^−^) but not mature HEL-specific B cells from sHEL donors survived and proliferated upon transfer to HEL-expressing recipients. Thus, T cell help appeared to be effective for only a limited time after antigen recognition. Since the life-span of B cells after antigen exposure in the absence of help is inversely related to the avidity of antigen binding [20, 21], we postulated that the window of opportunity for T cell rescue should also be an inverse function of BCR signal strength. Consistent with this hypothesis, IgM^+^ IgD^−^ CD23^−^ immature B cells from membrane HEL (mHEL) double Tg donors could not be induced to proliferate *in vivo*, although they were responsive to T cell signals when co-cultured with helper T cells *in vitro*, presumably due to a faster interaction between T and B cells.

## 2 Results

### 2.1 Experimental model for rescue of self-reactive B cells *in vivo*

**Figure 1:**
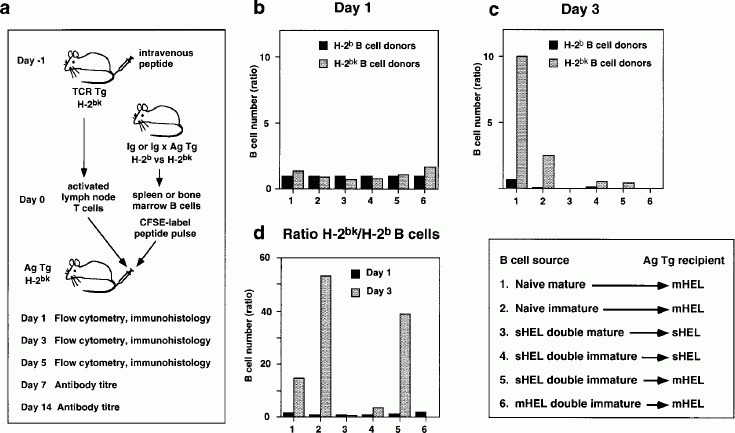
Summary of adoptive transfer experiments. (a) Experimental protocol for rescuing self-reactive B cells by means of antigen-specific T cell help. (b) Relative number of splenic H-2^bk^ (gray bars) and H-2^b^ (black bars) B cells 1 day after adoptive transfer. Values are normalized to the number of H-2^b^ B cells detected in the spleen on day 1 for each particular experiment. (c) Relative number of H-2^bk^ (gray bars) and H-2^b^ (black bars) splenic B cells 3 days after adoptive transfer. Values are normalized to the number of H-2^b^ B cells on day 1. (d) Ratio of H-2^bk^ to H-2^b^ splenic B cells 1 day (black bars) and 3 days (gray bars) after adoptive transfer. The experiments summarized in (b–d) are numbered according to the order in which they are described in the text.

It was shown previously that naive anti-HEL Tg B cells destined to become tolerant upon exposure to sHEL *in vivo* could be rescued and induced to differentiate and secrete antibody by provision of T cell help in the form of activated T cells from H-2^bk^ TCR Tg mice specific for moth cytochrome *c* peptide 87–103 (MCC_87–103_) in the context of I-E^k^ [12]. To control for possible antigen nonspecific effects of co-transferring activated T cells, all recipients were given activated H-2^bk^ T cells, together with either purified H-2^bk^ B cells with the capacity to present the appropriate peptide, H-2^b^ B cells that could not do so, or a mixture of the two (Fig. 1a). For focussing peptide to B cell MHC, one of two methods of comparable efficacy were used, *i.e. in vivo* administration of a fusion protein expressing both HEL and MCC_87–103_ epitopes (HELcyt [15]), or pulsing of purified B cells with MCC_87–103_ *in vitro* before transfer [12].

### 2.2 T cells rescue naive mature B cells from deletion by high-avidity self antigen

**Figure 2:**
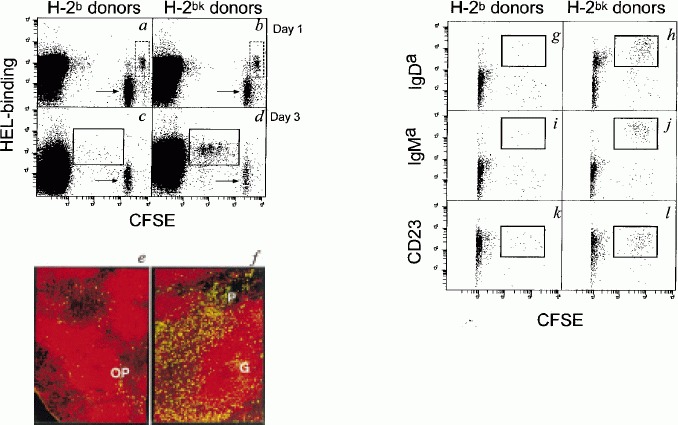
Both mature and immature Ig Tg B cells survive and proliferate when provided with T cell help upon transfer into mHEL Tg recipients. (a–d) FACS analysis of B220^+^ spleen cells obtained from mHEL Tg recipients of mature H-2^b^ (a, c) or H-2^bk^ (b, d) B cells. Undivided (dotted rectangles) and divided (solid rectangles) HEL-binding B cells are indicated. Syngeneic non-Tg B cell controls were quarter-labeled with CFSE (arrows). (e, f) Fluorescence micrographs of sections of mHEL Tg spleen 1 (e) and 5 (f) days after transfer of mature H-2^bk^ B cells. IgM^a^ B cells are green (FITC) and B220^+^ cells are red (Texas red). The outer PALS (OP), germinal centers (G) and proliferative foci (P) are indicated. (g–l) FACS analysis of B220^+^ spleen cells obtained from mHEL Tg recipients 3 days after transfer of immature bone marrow-derived H-2^b^ (g, i, k) or H-2^bk^ (h, j, l) B cells. The majority of recipient CFSE^−^ cells were excluded from collection to increase the sensitivity of detection of CFSE^+^ donor-derived cells. Cells that had divided and acquired a mature phenotype are indicated (solid rectangles).

The peptide-pulsing method was selected to perform a stringent test of the capacity of T cell help to rescue B cells from deletion. Mature splenic B cells from H-2^b^ or H-2^bk^ donors were pulsed *in vitro* with MCC_87–103_ and transferred together with activated H-2^bk^ TCR Tg T cells into H-2^bk^ mHEL Tg recipients. B cells were pre-labeled with carboxyfluorescein succinimidyl ester (CFSE) [22] to track cell division *in vivo*. Equal numbers of undivided anti-HEL Tg B cells were identified in recipient spleens one day later (Fig. 2a, b dotted boxes; Fig. 1b). By day 3, H-2^bk^ B cells had survived and proliferated in both the spleen (Fig. 2d solid box; Fig. 1c) and peripheral lymph nodes (not shown), whereas the majority of H-2^b^ B cells had disappeared (Fig. 2c solid box; Fig. 1c). Most HEL-specific B cells had divided four to six times between days 1 and 3, as indicated by their CFSE profile (Fig. 2d).

The small background of divided, HEL-binding H-2^b^ B cells (Fig. 2c solid box) probably represents cells which had proliferated in response to T cell help specific for B cell surface antigen/MHC complexes other than MCC_87–103_/I-E^k^. To rule out the possibility that bystander help stimulated by H-2^bk^ B cells could rescue H-2^b^ B cells even in the absence of direct T to B cell contact, CFSE-labeled, peptide-pulsed H-2^b^ B cells were transferred into sHEL recipients, with or without peptide-pulsed H-2^bk^ B cells. Equivalent numbers of H-2^b^ B cells were present in both groups of recipients on day 3 (23-fold and 15-fold fewer, respectively, than the number of control H-2^bk^ B cells, not shown).

Consistent with earlier data from sHEL recipients [21], immunohistology revealed that H-2^bk^ B cells had undergone arrest in the outer periarteriolar lymphoid sheath (PALS) of the spleen on day 1 (Fig. 2e), where they proliferated for several days before differentiating into proliferative foci and germinal centers (Fig. 2f) [12, 21]. The response was accompanied by production of anti-HEL antibody (mean serum concentrations of 13 μg/ml in recipients of H-2^bk^ B cells versus < 10 ng/ml in recipients of H-2^b^ B cells on day 14 after transfer). Thus, the short-lived pulse of T cell help provided by peptide-activated T cells i.v. and focussed by means of loading the B cells with peptide prior to adoptive transfer was sufficient to drive mature B cells to differentiate fully, even in the presence of high-avidity self antigen.

### 2.3 T cells rescue naive immature B cells from deletion by high-avidity self antigen

The fate of immature anti-HEL Tg B cells in the same experimental system was investigated using bone marrow cells depleted of mature cells on the basis of expression of IgD and CD23. Once again B cells from either H-2^b^ or H-2^bk^ Tg donors were CFSE labeled, pulsed with MCC_87–103_ and transferred with primed TCR Tg T cells into mHEL Tg recipients. Both B cell populations underwent several rounds of spontaneous cell division, as expected from previous studies in which immature B cells were transferred into non-Tg or sHEL recipients in the absence on T cell help [12]. However, the H-2^b^ donor B cells also underwent simultaneous deletion and the very few cells remaining by day 3 (Figs. 1c; 2g, i, k) still showed down-regulation of IgM and IgD despite having acquired CD23. Immature H-2^bk^ B cells that could respond to T cell help survived and by day 3 had assumed a mature phenotype (IgM^Hi^ IgD^hi^ CD23^+^) in the presence of multivalent self antigen (Fig. 2h, j, l, solid boxes). Nevertheless, in contrast to their mature counterparts (Fig. 2f), immature B cells did not undergo differentiation into effector populations visible on immunohistology (not shown), and made no serum antibody.

The division-dependent increase in B cell numbers in response to T cell help was 2.5-fold between days 1 and 3, compared with 10-fold for mature B cells in the previous experiment (Fig. 1c). On the other hand, the relative difference between the numbers of H-2^bk^ and H-2^b^ B cells on day 3 was actually greater (53-fold for immature cells versus 15-fold for mature cells, Fig. 1d), principally because immature H-2^b^ B cells died more rapidly than mature cells when exposed to mHEL in the absence of T cell help.

### 2.4 Effect of T cell help on B cells previously exposed to soluble self antigen *in vivo*

**Figure 3:**
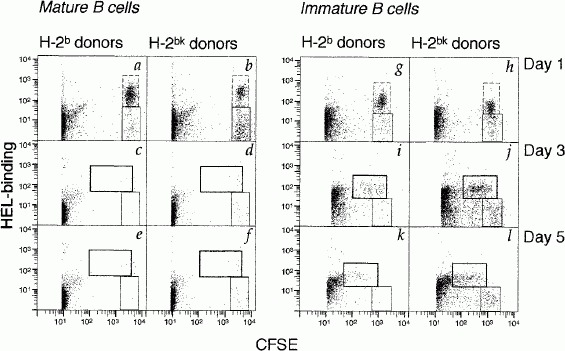
Rescue of immature but not mature self-reactive B cells from sHEL/anti-HEL double Tg donors. FACS analysis of B220^+^ spleen cells obtained from sHEL Tg recipients of mature (a–f) or immature (g–l) B cells at 1 (a, b, g, h), 3 (c, d, i, j) and 5 (e, f, k, l) days after transfer. The majority of recipient CFSE^−^ cells were excluded from collection to increase the sensitivity of detection of CFSE^+^ donor-derived cells. Undivided (dotted rectangles) and divided (rectangles with heavy borders) HEL-binding B cells are indicated. In addition, the proportion of surviving undivided donor-derived B cells that have down-regulated HEL-binding (rectangles with fine borders) is increased by T cell help, a phenomenon that is also apparent *in vitro* (not shown).

The above data showed that naive immature as well as mature B cells were deleted upon exposure to high-avidity antigen, unless rescued by simultaneous provision of T cell help. However, they left unanswered the question of whether T cell help could prevent deletion of B cells activated by previous exposure to self antigen, as may occur during normal B cell development *in vivo.* Initially MCC_87–103_-pulsed anti-HEL Tg B cells from the spleens of double Tg mice co-expressing sHEL were transferred together with T cell help into sHEL Tg recipients. Consistent with previously published data [16], no rescue of such mature tolerant B cells was seen (Fig. 3a–f). We hypothesized that the time between BCR ligation in the bone marrow and provision of T cell help 3–4 days later in the periphery was too long to allow reversal of the tolerant state [20, 23]. Accordingly, the impact of T help provided shortly after the BCR stimulus was investigated by substituting purified immature (IgM^+^ IgD^−^ CD23^−^) bone marrow B cells from sHEL-expressing double Tg mice for mature B cells. When tolerant immature B cells were provided with T cell help in the presence of soluble self antigen, a significant proportion survived, proliferated and acquired a mature phenotype (Figs. 1c; 3g–l). Although the number of cell divisions in the surviving HEL-specific B cells on day 3 was similar for both naive (Fig. 2j) and tolerant (Fig. 3j) immature B cells, the number of cells on day 3 was only 53 % of the number on day 1 (Fig. 1c), indicating that rescue of tolerant immature B cells was five foldless efficient than that of naive immature cells. Moreover, the ratio of B cells from H-2^bk^ versus H-2^b^ double Tg donors was only 3.6 (Fig. 1d), due to a combination of less efficient rescue of H-2^bk^ B cells and significantly increased survival of immature H-2^b^ B cells in sHEL versus mHEL recipients. Once again, no differentiation to germinal centers or proliferative foci was seen on immunohistology (not shown). Interestingly, provision of T cell help increased the proportion of divided and undivided B cells that had lost the capacity to bind HEL, which suggests that such cells changed their antigen specificity while in receipt of T cell help.

### 2.5 Prolonged T cell help fails to drive immature B cells to antibody production *in vivo*

**Figure 4:**
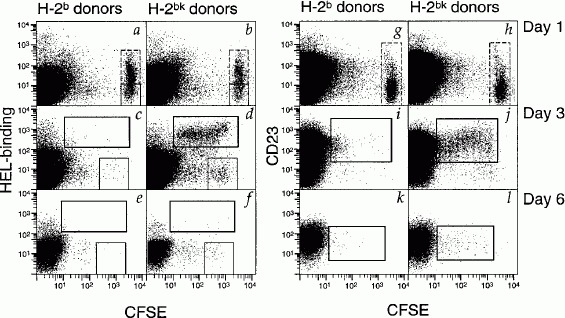
Rescue of immature self-reactive B cells transferred to mHEL hosts. FACS analysis of B220^+^ spleen cells obtained from mHEL Tg recipients of immature B cells from sHEL/anti-HEL double Tg donors at 1 (a, b, g, h), 3 (c, d, i, j) and 6 (e, f, k, l) days after transfer. Undivided (dotted rectangles) and divided (rectangles with heavy borders) HEL-binding B cells are indicated. Undivided donor-derived B cells that have down-regulated HEL-binding are indicated by rectangles with fine borders.

Previous *in vitro* experiments have indicated that immature B cells are capable of differentiating into antibody-producing cells in response to T cell help [24, 25]. To formally test whether peptide-pulsing provided too brief a T cell stimulus to drive immature B cells to a stage at which they could differentiate *in vivo*, the experiment was repeated using HELcyt [15] in addition to peptide pulsing, with a view to ensuring continuous presentation of the MCC_87–103_ epitope by HEL-specific H-2^bk^ B cells. Purified immature IgM^+^ IgD^−^ CD23^−^ bone marrow B cells from sHEL-expressing double Tg mice were pulsed with peptide and transferred together with activated T cells into mHEL recipients, followed by i.v. administration of HELcyt on days 0, 3 and 6. Once again, a cohort of immature tolerant B cells survived and proliferated in response to T cell help (Figs. 1c; 4a–l), showing very similar levels of early rescue to the previous experiment (41 % recovery on day 3), and a similar CFSE profile. The increase in the ratio of H-2^bk^ to H-2^b^ B cells on day 3 (Fig. 1d, comparable to that seen when naive immature B cells were transferred to mHEL hosts in exp. 2), resulted from the reduction in survival of H-2^b^ B cells in mHEL compared to sHEL hosts (see above). Consistent with the findings following transfer of naive immature B cells to mHEL hosts (exp. 2, not shown), no HEL-binding H-2^bk^ B cells were detectable on day 6 (Fig. 4l), indicating that the fate of immature B cells in mHEL hosts was not affected by previous exposure to sHEL nor by addition of HELcyt antigen. No evidence for differentiation was seen on immunohistology (not shown), nor was anti-HEL IgM^a^ antibody detected on day 6 or 14 after transfer.

The adoptive transfer was again repeated to provide activated T cell help over a longer period. In addition to repeated immunization with HELcyt, additional activated T cells were adoptively transferred on days 2 and 5. Once again, divided HEL-specific H-2^bk^ but not H-2^b^ B cells were seen on day 3 but had disappeared by day 6 (not shown).

### 2.6 Failure to rescue immature B cells previously exposed to high-avidity self antigen *in vivo*

**Figure 5:**
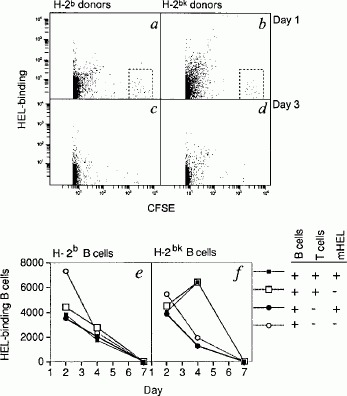
Immature self-reactive B cells from mHEL/anti-HEL double Tg donors respond to T cell help *in vitro* but not *in vivo.* (a–d) FACS analysis of B220^+^ spleen cells obtained from mHEL Tg recipients 1 (a, b) and 3 (c, d) days after transfer of immature bone marrow-derived B cells from mHEL/anti-HEL double Tg donors. Undivided immature B cells (dotted rectangles) have down-regulated surface Ig in response to mHEL. (e, f) *In vitro* response of immature B cells from mHEL/anti-HEL Tg donors co-cultured with activated T cells and either mHEL, sHEL or no HEL. Cells were harvested 2, 4, or 7 days after initiation of culture and HEL-binding B cells were counted by flow cytometry and expressed per 10^5^ live cells.

The data so far suggested that immature B cells developing in the presence of the low-avidity ligand, sHEL, could be rescued from deletion if T cell help was provided sufficiently early after the antigen signal. Since the life-span of B cells is an inverse function of the strength of the BCR-mediated signal, we tested whether immature B cells exposed to a high-avidity mHEL signal *in vivo* could also be rescued by T cell help. The previous experiment was therefore repeated except that immature B cells derived from the bone marrow of double Tg donors expressing mHEL were transferred into mHEL Tg recipients. T cell help had little impact on B cell survival, few cells being detectable in either the periphery or bone marrow by day 3 following transfer (Figs. 1c; 5a–d). To investigate whether this reflected an intrinsic inability of tolerant B cells from mHEL mice to respond to helper T cell signals, or a failure to attract T cell help sufficiently quickly following adoptive transfer, immature B cells from the same mHEL double Tg donors were pulsed with MCC_87–103_ and cultured *in vitro* with primed TCR Tg T cells in the presence or absence of mHEL-expressing thymocytes as a source of B cell antigen [26]. Under these circumstances, T cell help induced transient B cell expansion (Fig. 5e, f), clearly excluding an intrinsic defect in the ability of these cells to respond to T cell help as the primary reason for their failure to differentiate fully.

## 3 Discussion

The results described here and previously in the HEL Tg model indicate that the decision between deletion and survival of both immature and mature B cells is crucially dependent upon the provision of T cell signals within an appropriate period following recognition of antigen. Thus, provision of T cell help to both mature and immature naive self-reactive B cells can result in proliferation and rescue from early deletion in response to either a low- [12] or high- (Figs. 1, 2) avidity BCR-mediated signal. When a low-avidity signal has already been delivered in the bone marrow via soluble antigen, the life-span of the cells is reduced from approximately 4 weeks for naive B cells to only a few days [27]. Nevertheless, a proportion of immature IgM^+^ IgD^−^ CD23^−^ B cells purified from sHEL double Tg bone marrow can be rescued by T cell help *in vivo.* More mature cells that have already emigrated to the periphery cannot be rescued in the same experimental model. Our interpretation is that the most immature cells, *i.e.* those which have recognized antigen most recently, can still respond positively to helper T cell signals. Preliminary experiments involving the transfer of naive mature B cells into recipients expressing low-avidity sHEL indicate that rescue of mature B cells is not compromised if transfer of activated T cells is delayed by 24 h (E. Chan, B. Fazekas de St. Groth and A. Basten, unpublished data).

Receipt of a higher-avidity signal in the bone marrow shortens the average life-span of tolerant B cells from 3–4 days to less than 24 h [26, 27], allowing insufficient time for T cell help to act in the transfer system used here (Figs. 1, 5). Nevertheless the tolerant B cells can still be stimulated to divide *in vitro*, presumably because the physical proximity of T and B cells reduces the average time before T-B interaction occurs (Fig. 5).

Interestingly, provision of T cell help to naive or tolerant immature B cells increased the numbers of B cells that neither bound HEL nor expressed IgM^a^, suggesting that T cell signals afforded the B cells an opportunity of changing their specificity. While these data are reminiscent of the recent findings of Lang et al. [28], who showed that expression of bcl-2 in immature B cells increased the numbers of cells undergoing receptor editing, the correlation between lack of HEL specificity and lack of IgM^a^ expression was surprising, since the usual result of receptor editing [29, 30] is to modify the Ig specificity by rearranging a second light rather than heavy chain. It is tempting to speculate that the mechanism of immature B cell survival and differentiation in response to T cell help is mediated by up-regulation of genes such as bcl-2 and bcl-x_L_.

Our data, taken together with a number of published studies, indicate that the form of T cell help is a crucial regulator of the fate of tolerant B cells which have already been exposed to self antigen. For example, in our experiments in which T cells were activated by i.v. peptide 1 day before transfer, and in those of Rathmell et al. [17, 31], who used naive HEL-specific Tg T cells as a source of help, mature tolerant B cells could not be rescued upon transfer to sHEL hosts. On the other hand, when T cell help was provided by alloreactive T cells [16, 19] or normal T cells primed multiple times with HEL/CFA [18], mature tolerant B cells survived and differentiated to secrete anti-HEL IgM^a^, provided they also received a strong antigen signal (mHEL or HEL/CFA, respectively).

Previous studies in the HEL Tg model have pointed to involvement of the Fas and CD40 pathways in both T cell help and T-dependent deletion of B cells following BCR ligation, a phenomenon that can be detected only when the life-span of mature tolerant B cells is extended by transfer into non-Tg rather than sHEL hosts (M. Cook, A. Basten and B. Fazekas de St. Groth, unpublished results, and [17, 31, 32]). Our data support the conclusion that provision of T cell help immediately after BCR ligation promotes proliferation, whereas delayed help for tolerant B cells promotes deletion. In addition, comparison of the efficiency of rescue of naive sHEL double and mHEL double immature B cells provides evidence that the window of opportunity during which Fas- and CD40-mediated signals promote a combination of proliferation and survival [32] is an inverse function of the initial strength of the BCR signal.

These findings collectively underline the essentially passive role of B cells in the decision between tolerance and immunity, and suggest that the generation of self tolerance in the bone marrow may simply be a consequence of maturation in a T cell-deficient environment, rather than an intrinsic propensity of immature B cells themselves. Thus, the experimental evidence presented here is more consistent with the Bretscher-Cohn two-signal model of B cell tolerance and immunity than the Burnet-Lederberg hypothesis, which states that tolerance is the obligatory outcome of antigen recognition by immature B cells. Although immature B cells failed to differentiate fully in the experiments described here, previous data from Metcalf and Klinman [24, 25] have demonstrated that these cells as well as mature B cells could be driven to antibody production *in vitro.* Thus, complete differentiation in our *in vivo* experiments may have been compromised by technical factors relating to the phenotype of T cell help provided by T cells activated by i.v. peptide immunization, which are known to produce lymphokines for a maximum of 2 days (B. Fazekas de St. Groth, M. Wikstrom, A. Smith and L. Girgis, submitted for publication). In other words, the duration of help required to stimulate an autoantibody response from mature, self-reactive B cells, is very short. Further experiments using different forms of T cell help will be required to clarify this issue.

## 4 Materials and methods

### 4.1 Mice

All mice were bred and housed under specific pathogen-free conditions in the Centenary Institute Animal Facility. The following Tg mice were used: sHEL (ML5 line), in which the HEL transgene is under the control of the mouse metallo-thionein promoter [11]; mHEL (KLK3 line) which carries a transgene encoding HEL linked to the transmembrane region and cytoplasmic tail of MHC class I [33]; anti-HEL Ig bearing the IgH^a^ allotype (MD4 line [11]); anti-MCC TCR (-D line [34]). To allow collaboration between T and B cells to occur via presentation of MCC_87–103_ in association with I-E^k^, lymphocyte donors and recipients consisted of H-2^bk^ heterozygotes derived from crossing HEL or anti-HEL Tg mice on a C57BL/6 (IgH^b^ allotype) background with B10.BR mice, and TCR Tg mice on a B10.BR background with C57BL/6 mice. Anti-HEL Tg mice on a C57BL/6 background served as a source of control H-2^b^ B cells that did not present MCC_87–103_ to TCR Tg T cells.

### 4.2 Cell purification and adoptive transfers

Activated T cells were obtained by priming H-2^bk^ TCR Tg mice with an i.v. injection of 10 μg MCC_87–103_ 20–24 h before isolation of lymph node cells. This resulted in activation of >80 % of peptide-specific CD4^+^ T cells. H-2^bk^ or H-2^b^ B cell preparations were depleted of T cells with a mixture of rat IgM anti-CD4 (RL172.4 [35]), anti-CD8 (3.155 [36]), and anti-Thy 1 (HO13.4 [37]) followed by young rabbit complement (C-six Diagnostics, Mequon, WI). After fractionation on discontinuous Percoll gradients (Pharmacia, Uppsala, Sweden), cells at the 65/70 % interface were pooled for adoptive transfer. Bone marrow suspensions were depleted of mature (IgD^+^ CD23^+^) B cells using a mixture of AMS15.1-biotin [38] and B3B4-biotin [39] followed by streptavidin-conjugated Dynabeads (Dynal, Oslo, Norway). The average percentage of B220^+^ cells in the depleted bone marrow was 8 % and the degree of residual contamination by mature B cells was less than 0.02 % in all experiments.

For pulsing with cytochrome peptide, purified B cells were resuspended at 5 × 10^7^/ml and incubated with 10 μM MCC_87–103_ for 2 h at 37 °C, then washed three times before adoptive transfer. Cells were fully or quarter labeled with 5-carboxyfluorescein diacetate succinimidyl ester (CFSE; Molecular Probes, Eugene, OR) as described previously [12]. Adoptive transfers were performed by i.v. injection into the lateral tail vein of unirradiated H-2^bk^ HEL Tg recipients. B220^+^ B cells (10^6^ of either mature or immature cells) were injected together with 10^6^ activated T cells per recipient. In exps. 1–4 and 6, two recipients were analyzed at each of four time points, whereas twice that number were used in exp. 5.

Immunization with HELcyt, a fusion protein of HEL and MCC expressed in the baculovirus system [15], was performed by three i.v. injections of 50 μg antigen on days 0, 3 and 6 after adoptive transfer.

### 4.3 Flow cytometric analysis

Four color flow cytometry was performed on a FACStar^Plus^ (Becton Dickinson, Mountain View, CA) and analyzed using CellQuest (Becton Dickinson) software. Surface antigens were identified with the following reagents: B220, RA3-6B2-phycoerythrin (PE) (Caltag, S. San Francisco, CA); IgM^a^, RS3.1-biotin followed by stretavidin-allophycocyanin (APC) (Molecular Probes); IgD^a^, AMS15.1-biotin followed by streptavidin-APC; CD23, B3B4-biotin (PharMingen, San Diego, CA) followed by streptavidin-APC. HEL-binding B cells were identified as described previously [12] using a three-layer stain comprising HEL (Sigma, St. Louis, MO), HyHEL-5-biotin and streptavidin-APC. Propidium iodide was included in all samples and non-viable cells were excluded from analysis.

### 4.4 Immunohistology

Fragments of spleen were snap frozen in liquid nitrogen. Frozen sections of spleen (5 μm) were thaw-mounted onto glass slides and fixed and stained as described previously [12]. Anti-HEL Tg B cells were identified with a combination of RS3.1-FITC and RA3.6B2 followed by anti-rat Texas red (Caltag). After the final wash, slides were wet mounted and photomicrographs were obtained using a Leitz DMR BE fluorescence microscope (Wetzlar, Sweden).

### 4.5 B cell cultures

CFSE-labeled immature B cells (1 × 10^6^) from either H-2^bk^ or H-2^b^ donors and 1 × 10^6^ H-2^bk^ T cells were cultured at 37 °C in 5 % CO_2_ for the times indicated in the Sect. 2.6. Cultures contained either sHEL (200 ng/ml), mHEL (5 × 10^5^ thymocytes from mHEL Tg donors) or no antigen in 2 ml RPMI 1640 supplemented with 10 % fetal calf serum (Commonwealth Serum Labs, Melbourne, Australia), 0.01 M sodium bicarbonate, 50 μg/ml penicillin, 100 μg/ml streptomycin and 5 × 10^−5^ M 2-mercaptoethanol.

## Abbreviations

BCR: B cell receptor
CFSE: Carboxyfluorescein succinimidyl ester
HEL: Hen egg lysozyme
HELcyt: Fusion protein of HEL and MCC_87–103_
MCC_87–103_: Tobacco hornworm moth cytochrome c peptide 87–103
PALS: Periarteriolar lymphoid sheath
Tg: Transgenic
